# SBML and MathML support for the stochastic reaction-diffusion simulator STEPS

**DOI:** 10.1186/1471-2202-12-S1-P57

**Published:** 2011-07-18

**Authors:** Iain Hepburn, Weiliang Chen, Erik De Schutter

**Affiliations:** 1 Computational Neuroscience Unit, OIST, Okinawa, 904-0411 Japan; 2 Theoretical Neurobiology, University of Antwerp, B-2610 Antwerpen, Belgium

## 

STEPS is a stochastic signaling pathway simulator based on the “spatial Gillespie” method for reaction-diffusion simulation, within a Python interface [1]. STEPS supports tetrahedral meshes, which allow for complex boundary representation absent from simulators based on cubic voxels. A powerful addition to STEPS, the membrane potential calculation, is under development. The potential of nodes throughout the tetrahedral mesh are simulated, which allows for the inclusion of voltage-gated membrane channels with molecular channel currents within the simulation.

STEPS supports spatial and non-spatial simulations, and future versions will even allow for a combination of spatial and well-mixed compartments in one simulation. The Systems Biology Markup Language (SBML) is a widely used format for representing models of networks of biochemical interactions. The neuronal signaling pathways that STEPS is designed to simulate in a well-mixed context fall under this umbrella.

While it was found that the Python interfaces to STEPS and libSBML makes it an ease to support most components of SBML, in some models there may be hurdles that cannot be overcome in a STEPS context and also any other stochastic simulation context. Stochastic simulation approaches, including the SSA that STEPS is based on, assumes that each reaction is the most fundamental with no intermediate, non-simulated steps. Therefore the expected form of the mathematics for the reaction rate is easily definable, but may differ from the explicit mathematics specified in some SBML files. If STEPS were to ignore alternate reaction kinetic law mathematics then any model that included such would be represented incorrectly. An example is a catalyst reaction that may often be described as a single reaction whose kinetic mathematics is influenced by the catalyst, which should typically be broken down into three or more separate reactions in the stochastic description.

STEPS performs a comparison of the reaction kinetic law mathematics to the form that is expected, which means that a model will never be simulated incorrectly. This is achieved by converting the MathML components in SBML, including reaction kinetic law mathematics, to Python objects with no limit on depth. MathML support in the Python interface then makes it easy to support other features of SBML that are based on MathML, which are numerous. This is because the MathML Python objects are available during simulation and may reference any variable in the model including those whose value is not constant during simulation. This extends SBML support in STEPS to many components that other stochastic simulators cannot support because they ignore MathML, including important features such as Initial Assignments, Assignment Rules, Rate Rules and Events.
